# General method for emulsion polymerization to yield functional terpolymers

**DOI:** 10.1016/j.mex.2020.101110

**Published:** 2020-10-16

**Authors:** Alexandra J. Macbeth, Zhuangsheng Lin, Julie M. Goddard

**Affiliations:** a Department of Food Science, Cornell University, Ithaca, NY 14850, United States; b Department of Chemistry and Chemical Biology, Northeastern University, Boston, Massachusetts 02115, United States

**Keywords:** Emulsion polymerization, Films, Functional coatings, Bioactive materials, Chelating polymer, Copolymer synthesis

## Abstract

Copolymerization methods are used to impart specific, desired functional properties (e.g. mechanical or bioactive) to a material for targeted applications in biomedicine, food and agriculture, consumer products, advanced manufacturing, and more. Many polymerization methods exist to achieve tailored copolymer architectures. Of them, emulsion polymerization offers unique and industrially convenient features that make for easily scalable processes because the synthesis occurs in water and the latexes usually do not need further purification. Because of the breadth of copolymer architectures and thus wide range of potential applications for latexes produced by emulsion polymerization, there is great value in defining general methods for emulsion polymerizations to yield copolymers, including routes for synthesis of functional monomer building blocks, to permit consistency and optimization of these processes. Herein we present a general emulsion polymerization method for synthesis of a copolymer consisting of three functional monomers, suitable for adaptation to alternate base chemistries, curing chemistries, and functional ligands. This protocol includes the synthesis of the functional monomers glycidyl methacrylate-iminodiacetic acid (GMA-IDA) and 4-benzolylphneyl methacrylate (BPM).

• Our synthesized copolymer includes a glycidyl methacrylate (GMA) monomer functionalized with a metal-chelating iminodiacetic acid (IDA) ligand, a UV-curable monomer, 4-benzoylphenyl methacrylate (BPM), and an inert hydrophobic monomer, *n‑*butyl acrylate (BA).
• The presented synthesis route demonstrates a general polymerization method that can be modified to copolymerize alternative functional monomers to create multi-functional polymers.

## Graphical abstract

**Figure:**
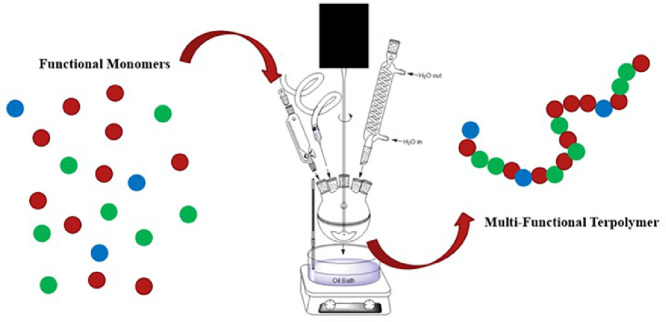

Specifications table

**Table:** 

Subject Area	Chemistry, Materials Science, Agricultural and Biological Sciences
More specific subject area	*Polymer Chemistry*
Method name	*General Emulsion Polymerization of Functional Copolymers*
Name and reference of original method	Lin, Z., & Goddard, J. M. (2018). Photocurable coatings prepared by emulsion polymerization present chelating properties. *Colloids and Surfaces B: Biointerfaces*, *172*, 143–151.
Resource availability	Mechanical Stirrer: available through ChemGlass Reactor Stand: https://chemglass.com/chemrxnhub-support-stands-benchtop-reactors?AspxAutoDetectCookieSupport=1 Glass stir rod: https://chemglass.com/stirrer-shafts-polished-10mm-chem-stir?sku=CG-2078–05 Agitator: https://chemglass.com/agitator-ptfe-anchor-style 2-part, 5-neck, 300 mL round bottom flask was ordered custom through ChemGlass. Personal Protective Equipment: Long pants, lab coat, closed toed shoes, eye protection (safety glasses) and gloves. Gloves can be natural rubber, butyl rubber, nitrile rubber, neoprene or polyvinyl chloride (PVC). Reagents: Purchased from SigmaAldrich and Fisher Scientific and used as received

## Method details

### Part I: Synthesis of monomer 1: glycidyl methacrylate – iminodiacetic acid (GMA-IDA)

#### Reaction duration

**Fig. 1:**
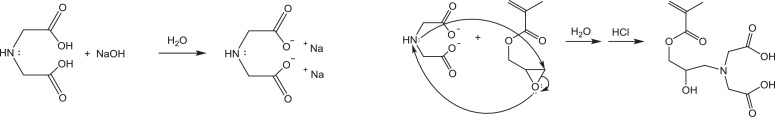
Mechanism for the synthesis of monomer 1: glycidyl methacrylate-iminidiacetic acid (GMA-IDA) beginning with a neutralization of iminodiacetic acid (left) followed by an epoxy ring opening to join GMA and IDA together (right).

3 h total: 0.5-hour synthesis preparation + 1-hour synthesis + 1.5-hour purification (Fig. 1)

#### Reagents

Sodium hydroxide pellets, deionized (DI) water, iminodiacetic Acid (IDA) (98+%), glycidyl methacrylate (GMA) (97%), concentrated hydrochloric acid (trace metal grade), and acetone.

NOTE: this general method can be adapted to synthesize other functional (e.g. antimicrobial, biotinylated) monomers using the glycidyl methacrylate base monomer.
NOTE: many hazardous reagents are used in this method; carefully review and post SDS (safety data sheets) for the safety of the individual performing the synthesis and others in the laboratory.

#### Equipment

The reactor consists of a 300 mL two-part, 5-neck round bottom flask, overhead mechanical stirrer, glass stirring rod and anchor style agitator, reactor stand, hot plate, battery operated thermometer with clip, oil bath set to 65 °C, condenser, addition funnel, 3 rubber septum stoppers. Other equipment includes  250 mL beaker, magnetic stir bar and stir plate, parafilm, 10 mL mechanical pipette, 5 mL mechanical pipette, ring stand with ring attachment compatible with 500 mL separatory funnel, 500 mL separatory funnel, clean Erlenmeyer flasks for extraction, Buchner funnel, filter paper, and a vacuum line.

NOTE: the nitrogen line is not necessary for Part I, however it will be utilized later in Parts II and III.

#### Preparation

**Fig. 2:**
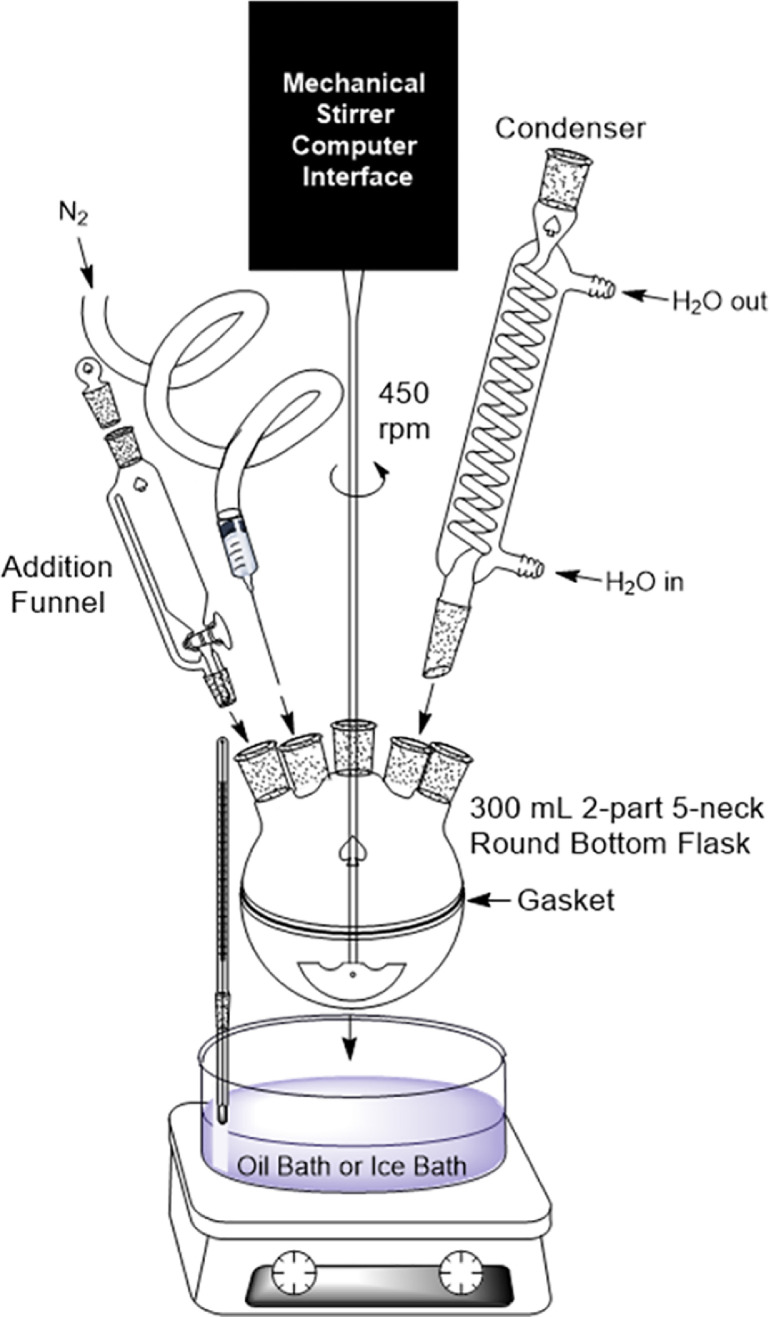
Diagram for the 300 mL reactor set-up. Note the option for an oil bath or an ice bath.

1. Assemble the reactor as shown in Fig. 2, but without the nitrogen line. Plug the three, free necks with rubber septum stoppers. Heat the oil bath to 65 °C.

**Fig. 3:**
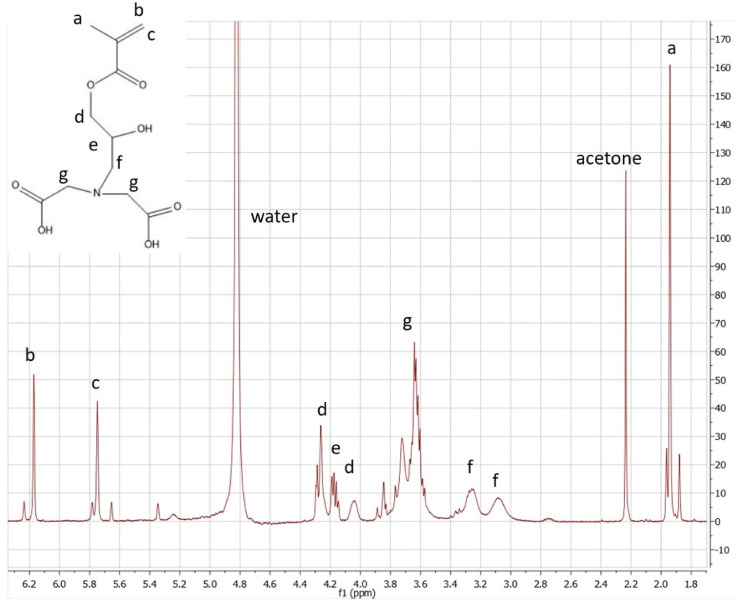
Proton NMR spectrum of 2-propenoic acid,2-methyl-,3-[*bis*-(*carboxymethyl*) *amino*]−2-hydroxypropyl ester (GMA-IDA) in D_2_O (400 MHZ). Reprinted from Colloids and Surfaces B: Biointerfaces, Vol. 172, Lin, Z., Goddard, J., *Photocurable coatings prepared by emulsion polymerization present chelating properties*, pp 143–151, 2018, with permission from Elsevier. To verify the product has the structure presented in Fig. 3, use proton NMR.

NOTE: the reactor is set up in a dedicated fume hood, and should only be performed by someone trained to safely conduct the synthesis.

1 Prepare a 50 mL solution of 2 M NaOH:
  i. In a 250 mL beaker equipped with a magnetic stir bar, add 4 g of NaOH pellets to 50 mL DI water.
  ii. Cover the beaker with Parafilm^Ⓡ^ and place on a magnetic stir plate set to 350 rpm. Let stir until all the NaOH is dissolved, about 10 min.
    TIP #1: Mixing NaOH into water is highly exothermic and will produce heat.
    TIP #2: It is recommended to perform this step in a secondary containment vessel in case of spills.

#### Procedure

1. Add 6.055 g of IDA to a solution of 50 mL of 2 M NaOH to achieve a final concentration of 0.91 M IDA. The beaker was covered with parafilm and left to stir at 350 rpm until fully dissolved, about 10 min.  The resulting product is ‘neutralized IDA’.
2. Add the 50 mL solution of neutralized IDA to the reactor and begin the mechanical stirring at 450 rpm using the overhead stir bar.
3. Using a 10 mL mechanical pipette, add 6.821 mL of GMA to the addition funnel. Then open the stopcock of the addition funnel slightly to add the reagents to the reactor at a rate of approximately 1 drop per second.  The solution will turn from colorless to white upon the addition of GMA.
  *TIP #3: Angle the tip of the funnel towards the center of the round bottom flask so the reagent is added directly to the center of the reaction, and to avoid losing reagent from rolling down the edges of the flask.*
4. After all the GMA has been added to achieve a final concentration of 0.91 M corresponding to a 1:1 molar ratio of GMA:IDA, replace the addition funnel with a rubber septum stopper and let the reaction proceed for 1 hour.
  *NOTE: The mixture will turn from colorless to white after the addition of GMA.*
5. Using a 5 mL mechanical pipette, neutralize the crude product with 2 mL of concentrated HCl.
  *TIP #3 applies: angle the pipette tip towards the center of the reaction to avoid losing acid on the sides of the round bottom*
6. Purify the GMA-IDA product by washing[1].
  i. Add the crude product along with 300 mL of acetone to a 500 mL separatory funnel. Gently shake the funnel, and release the stopcock to relieve pressure. Repeat this inversion process until there is no longer pressure buildup after inversion. In other words when there is no longer a sound upon of pressure-release.

TIP #2: Make sure to be gentle when shaking the separatory funnel as vigorous shaking may cause excess pressure to buildup.
  i. Secure the funnel using a ring stand and leave it to sit for 5–10 min to allow the inorganic and organic layers to separate.
  ii. Remove the stopper of the separatory funnel and open the stopcock slightly to slowly drain the bottom aqueous layer containing the GMA-IDA. Discard the top layer.
  iii. Repeat steps i-iii with 50 mL DI water keeping the aqueous layer and discarding the organic layer.
  iv. Repeat steps i-iv three more times plus an additional final wash with 300 mL acetone.
  v. Dry the product in a vacuum desiccator for 20 min at room temperature to remove excess acetone.
7. The final product should be a clear, slightly viscous liquid. Store at 4 °C until it is needed for the copolymerization in Part III.

*TIP #4:  Storage conditions should be altered appropriately for light or moisture sensitive monomers, for example to store in an amber vial or over desiccant.*

## Method validation

### Part II: Synthesis of monomer 3: 4-benzoylphenyl methacrylate (BPM)

#### Reaction duration

20 h + 3-day total: 0.5 h for synthesis preparation + 18-hour synthesis + 1.5 hour purification + 3-day dry

#### Equipment

The reactor consists of a 300 mL two-part, 5-neck round bottom flask, overhead mechanical stirrer, glass stirring rod and anchor style agitator, reactor stand, battery operated thermometer with clip, ice bath, funnel, nitrogen line and 3 rubber septum stoppers. Other equipment includes 10 mL syringe*,* 5 mL syringe*,* 2 sparging needles*,* stopcock compatible with syringe and needle*,* parafilm, scissors*,* aluminum foil, 200 mL graduated cylinder*,* 10 mL mechanical pipette*,* filter paper and a Buchner funnel.

#### Preparation

1. Assemble the reactor as shown in Fig. 2, including an addition funnel, nitrogen line and ice bath. Plug the three free necks with rubber septum stoppers.
  *NOTE: The ice bath is to prevent excess heat in the beginning of the reaction. It is not necessary to keep the ice bath cold throughout the duration of the reaction. If your specific reaction requires a cold ice bath for a long period of time, considering employing an immersion chiller probe. See link above in “Resource Availability.”*
  *TIP #5: make sure the agitator anchor is as close to the bottom of the round bottom flask as possible to ensure the homogenization of 4-hydroxylbenzophenone and diethyl ether.*
2. Purge the reactor and addition funnel with nitrogen and then plug the reactor and turn the flow off. You do not need a continuous flow of nitrogen throughout the reaction.
3. To prepare for air-free collection of methacryloyl chloride
  i. Cut a 10 mL syringe equipped with a stopcock at the 4 mL mark using scissors. Wrap Parafilm^Ⓡ^ around the cut end of the syringe to prepare it for an air tight seal with a rubber balloon. Place the mouth of a rubber balloon around the Parafilm^Ⓡ^ wrapped syringe and seal it as tight as possible with a plastic cable tie.
  ii. With the stopcock of the syringe open, fill the balloon with nitrogen and close off the stopcock. Connect a sparging needle to the end of the stopcock on the syringe and plunge it into the rubber top of the methacryloyl chloride ensuring that the tip of the needle remains in the air region of the bottle, above the reagent.
  iii. Plunge a 5 mL syringe equipped with a sparging needle into the rubber top of the reagent bottle, feeding the needle all the way down into the reagent. Open the stopcock on the syringe holding the nitrogen filled balloon to allow nitrogen to flow to replace the collected reagent. Use the 5 mL syringe to collect 5 mL of mathacryloyl chloride. Flip the syringe upside down while still in the reagent to rid the vessel of any air bubbles.

TIP #6: When handling methacryloyl chloride, wear gloves (natural rubber, butyl rubber, nitrile rubber, neoprene or polyvinyl chloride (PVC)), protective goggles and a respirator mask and work in the fume hood since this is a volatile and toxic reagent. Methacryloyl is flammable, toxic if inhaled and corrosive.

2 Prepare a 0.1% w/w NaOH solution using the method mentioned in step 2 of *Synthesis Preparation* in Part I.

#### Procedure

1. Using a 200 mL graduated cylinder, add 150 mL diethyl ether to the reaction vessel and set the overhead stirring to 450 rpm.
  *TIP #7: Diethyl Ether is a peroxide former - test your Diethyl Ether every 6 months for peroxides.*
2. Add 10.32 g of 4-hydroxybenzophenone to the reaction vessel and let homogenize for 10 min.
3. Using a 10 mL pipette, charge 7.985 mL of triethyl amine into the reaction vessel and then purge the reaction with nitrogen to achieve a 1:1 molar ratio of triethylamine:4-hydroxybenzophenone.
4. Cover the reaction vessel in foil to allow the reaction to proceed in a light-free environment since the product is light sensitive.
  *TIP #8: Wrap the foil in such a way to create an easy peel-back flap that allows for reaction monitoring if necessary.*
5. Add 25 mL of diethyl ether to the addition funnel.
6. To the diethyl ether filled addition funnel, add the previously collected 5 mL methacryloyl chloride.
  *TIP #9: The sparging needle was placed directly in the diethyl ether to add the methacryloyl chloride to ensure homogenization of the reagents.*
7. Open the stopcock of the addition funnel to allow the reagents to add dropwise to the reaction to achieve a 1:1:1 molar ratio of methacryloyl chloride:triethylamine:4-hydroxybenzophenone. The solution should appear white and liquid. Afterwards, replace the addition funnel with a rubber stopcock and let reaction was proceed in the dark for 18 h.
  *TIP #3* again applies here.
8. After the completion of the reaction, filter off the triethylammonium hydrochloride precipitate using filter paper and a glass funnel.
  *NOTE: The product should be a white liquid, not a yellow solid. If the resulting product is solid, it is most likely there was a significant leak in the reactor causing the diethyl ether fumes to evaporate.*
  *TIP #10: Carry out all post-synthesis steps in as low-light an environment as possible to since BPM is a light sensitive reagent. Use foil whenever possible.*
9. Wash the filtrate with 200 mL DI water and 200 mL 0.1% NaOH following the same extraction procedure mentioned in Part I, until no yellow color is observed in the organic phase.
10. Dry the product over MgSO_4_
  i. Using a metal scooper, add an excess of magnesium sulfate to the product collected in step 9.
  ii. Swirl the product between additions of magnesium sulfate.
  iii. Repeat with additions of magnesium sulfate until you see the *snowstorm* effect. This is when the magnesium sulfate is freely flowing and takes a while to settle to the bottom, similar to what is seen in a snow globe.
11. Evaporate the diethyl ether from the BPM using a vacuum and Buchner funnel. The resulting product should be a white solid.
12. Dry the product in a vacuum desiccator for 3 days to collect the BPM and store in a sealed foil-covered vessel in the refrigeration until further use.

#### Method validation

**Fig. 4:**
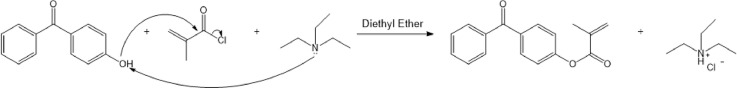
Mechanism for the synthesis of monomer 3: 4-benzoylphenyl methacrylate (BPM). *Reagents:* 4-hydroxybenzophenone (98%), diethyl ether, triethyl amine (99.5%), methacryloyl chloride (97%), DI water, sodium hydroxide pellets and magnesium sulfate.

To verify the product has the structure presented in Fig. 4, use proton NMR.

## Part III: Emulsion polymerization for the copolymer synthesis (poly(2-propenoic acid,2-methyl-,3-[bis-(carboxymethyl) amino]−2-hydroxypropyl ester-co-n‑butyl acrylate-co-4-benzoylphenyl methacrylate) (GMA-IDA-co-BA-co-BPM))

### Reaction Duration

27 h total: 0.5-hour synthesis preparation + 20-hour reaction + 6.5-hour product purification

### Reagents

Butyl acrylate (BA), potassium persulfate (KPS) (99+%), DI water, methanol, GMA-IDA synthesized from Part I and BPM synthesized from Part II.

TIP#11: In this example emulsion polymerization, reagents (including the monomer butyl acrylate) were used as received. Monomers can be purified by distilling under reduced pressure if desired and the  purity of reagents can be verified by NMR spectroscopy and additional purification steps [2].

### Equipment

The reactor consists of a 300 mL two-part, 5-neck round bottom flask, overhead mechanical stirrer, glass stirring rod and anchor style agitator, reactor stand, hot plate, battery operated thermometer with clip, oil bath set to 70 °C, condenser, nitrogen line and 4 rubber septum stoppers. Other equipment includes a 150 mL graduated cylinder, 10 mL mechanical pipette, 50 mL beaker, 1 mL mechanical pipette, and regenerated cellulose dialysis membranes.

### Preparation

1 Assemble the reactor as shown in Fig. 2, including the nitrogen line and oil bath heated to 70 °C. Plug the free necks with rubber septum stoppers.

### Procedure

1. Add 2.7 g of GMA-IDA and 0.39 g KPS to the reactor along with 126 mL of DI water to achieve a 10:1 molar ratio of GMA-IDA monomer:KPS initiator. Homogenize the reagents at a stir rate of 450 rpm.
2. Dissolve 2.4 g of BPM in 10.8 mL of BA and charge into the reaction vessel to yield a total monomer ratio of 7:1:1 BA:GMA-IDA:BPM.
3. Start the nitrogen flow to the reactor and bring the oil bath up to 70 °C. Cover the reactor with foil and the let it proceed for 20 h in the dark.
  *TIP #8 again applies here.*
4. The crude product should be a white liquid. Centrifuge the crude product at 3000 gs for 15 min to remove polymer sediments. Repeat twice.
5. Treat the supernatant with dialysis to remove the unreacted reagents:
  i. To remove the unreacted GMA-IDA
    a. Using scissors, cut 20 kDa dialysis membrane tubes according to the required volume per length listed on the packaging of the dialysis tubes.
      *TIP #12: Leave an extra 5–6* *cm of tubing to account for the clips that will be placed on either end.*
    b. Soak the tube in DI water for 10 min.
    c. Fold over one end of the tube and secure it with a clip and add the copolymer to the tube. Clip the other end.
      *TIP #13: Put the tube in a small beaker clip side down to catch spills. The tubing is quite flexible so this prevents likely loss of polymer*.
    d. Fill a 1 L beaker with DI water and place a magnetic stir bar at the bottom. Place the filled dialysis tube in the DI water, put the beaker on a magnetic stir plate and begin stirring at 350 rpm.
    e. Let the dialysis continue for 3 h.
  ii. To remove the unreacted BA and BPM:
    a. Repeat step i, a-e except substitute DI water for methanol.
6. Centrifuge the purified retentate twice more at 3000 gs for 15 min and collect the purified polymer supernatant and store in the refrigerator.

### Method validation

**Fig. 5:**
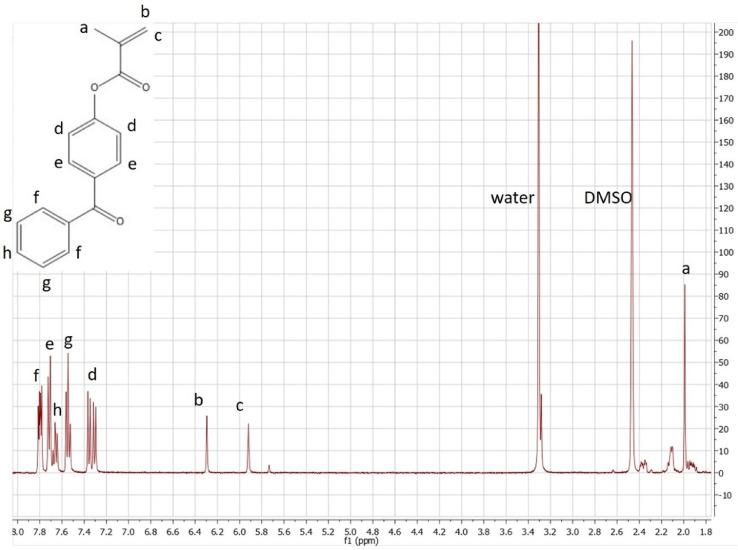
Proton NMR spectrum of 4-benzoylphenyl methacrylate (BPM) in DMSO‑d_6_ (400 MHZ). Reprinted from Colloids and Surfaces B: Biointerfaces, Vol. 172, Lin, Z., Goddard, J., *Photocurable coatings prepared by emulsion polymerization present chelating properties*, pp 143–151, 2018, with permission from Elsevier.

**Fig. 6:**
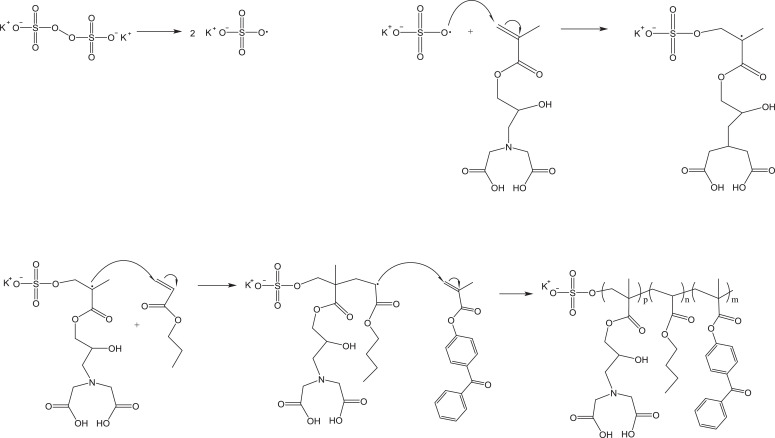
Mechanism for initiation and copolymerization via emulsion polymerization of monomers glycidyl methacrylate-iminodiacetic acid (GMA-IDA), butyl acrylate (BA), and 4-benzoylphenyl methacrylate (BPM). *NOTE:* This is a random copolymer, i.e. the monomers will add to the chain randomly and in unequal proportions. See Fig. 7.

To verify the product has the structure presented in Fig. 5, use proton NMR. To verify that the copolymer is curable via exposure to UV irradiation, monitor the absorption spectrum at 270–290 nm as the coating is being exposed to UV-light (365 nm). The absorption band at 270 nm should decrease as a result of successful benzophenone crosslinking as shown in Fig. 8. To verify the copolymer contained poly(n‑butyl acrylate) and GMA functionalized with IDA ligands, take ATR-FTIR and absorbance spectra of polypropylene coated with the copolymer compared to bare polypropylene as the control as shown in Fig. 9. Bare polypropylene should show absorption bands at 3000–2800 cm^-1^ for the C—H stretch and at 1450 cm^-1^ and 1370 cm^-1^ for the C—H bend. For the copolymer film, absorption band at 3000–2800 cm^-1^ for the C—H stretch and a strong absorption band at 1710 cm^-1^ for the carbonyl stretch and bands at 1260–1160 cm^-1^ for a ether stretch indicate poly(n‑butyl acrylate) is present while a small shoulder at shoulder at 1620 cm^-1^ indicate the presence of IDA ligands [3]. (Fig. 6)

**Fig. 7:**
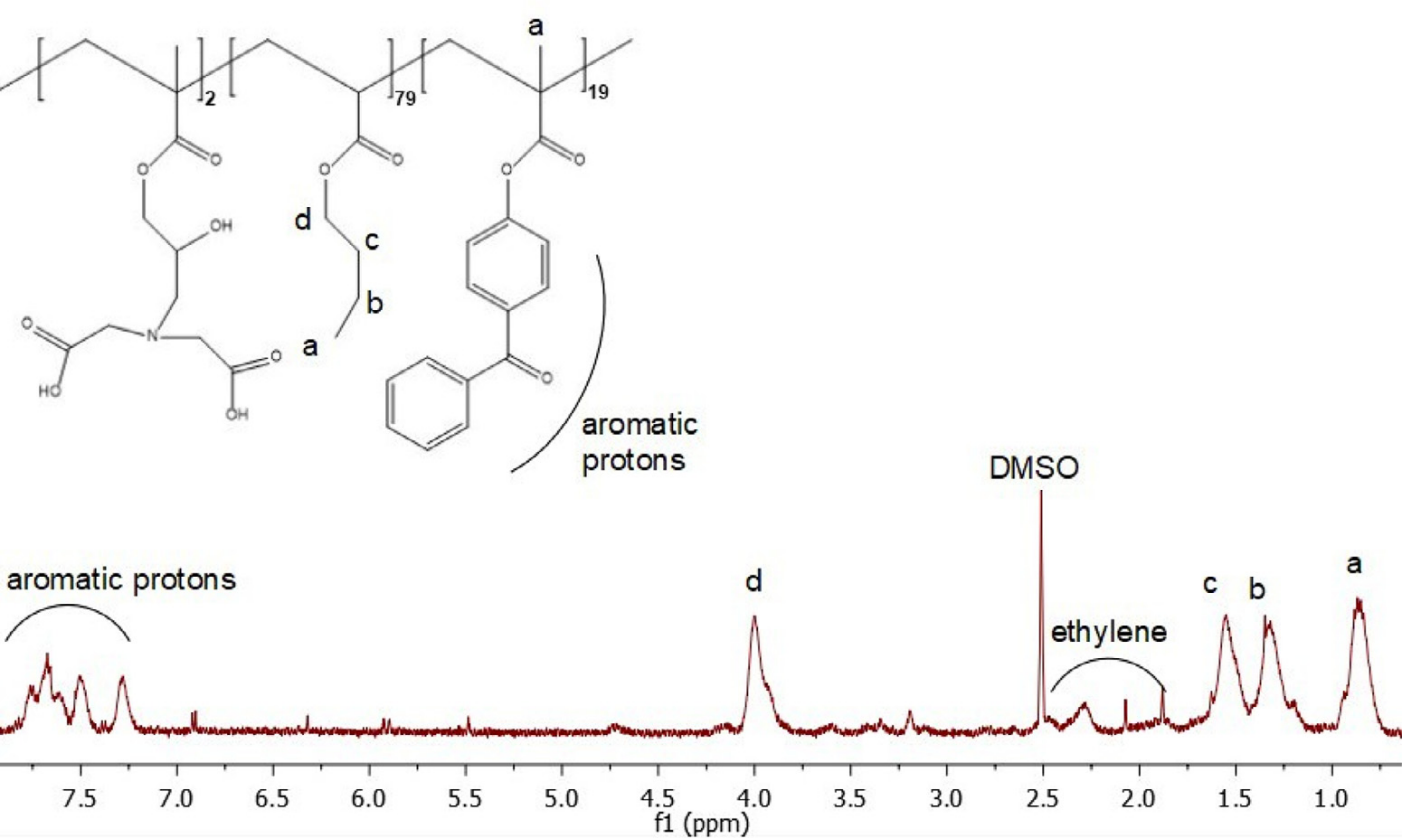
Proton NMR spectrum of GMA-IDA-co-BA-co-BPM polymer collected in DMSO‑d_6_ (130 °C, 600 MHZ). Reprinted from Colloids and Surfaces B: Biointerfaces, Vol. 172, Lin, Z., Goddard, J., Photocurable coatings prepared by emulsion polymerization present chelating properties, pp 143–151, 2018, with permission from Elsevier.

## Conclusion

The above method provides a detailed synthesis of a functional terpolymer via emulsion polymerization including details for the reactor set-up to ensure a controlled reaction environment. Thus, these methods provide a foundation and synthesis ‘tips and tricks’ that can be applied and adapted to generate copolymers with different functionalities utilizing emulsion polymerization methods. For example, thermocuring monomers, antimicrobial or non-fouling functional ligands, bio-based environmentally sustainable monomers, or biocompatible monomers with applications in food packaging, food safety, biomedical implants, or functional polymer coatings.

## Additional information

Copolymers enable combining the functional or mechanical properties of more than one homopolymer in a single material [4] making them useful in a wide array of applications that require multiple polymer characteristics such as clothing, rubbers, latex paints, materials for automobiles, electronics, furniture, construction materials, packaging, adhesives and biomedical applications [5]. For example, some polymer functionalities useful for applications in active packaging could include a metal chelating ligand that improves the food's shelf-stability and a UV-curable moiety that promotes its application in high-throughput industrial processes because they do not require solvents nor high energy inputs to cure [6], [7], [8].

There are many polymerization methods employed to achieve copolymer architectures, however emulsion polymerizations are advantageous because their resulting latexes do not generally need further purification making this a viable option for high-throughput processes and synthesis occurs in water, and thus is a more environmentally friendly process. Emulsion polymerizations proceed via radical polymerization and are widely used to synthesize industrial polymers for examples adhesives, paints, binders, textiles and construction materials [9]. Although there are strong advantages to emulsion polymerization, these syntheses generally involve complex reactor set-ups that require many steps and which can lead synthesis inconsistency[10]. Thus, standard methods for copolymer syntheses via emulsion polymerization are needed to improve the consistency and optimization of these processes.

**Fig. 8:**
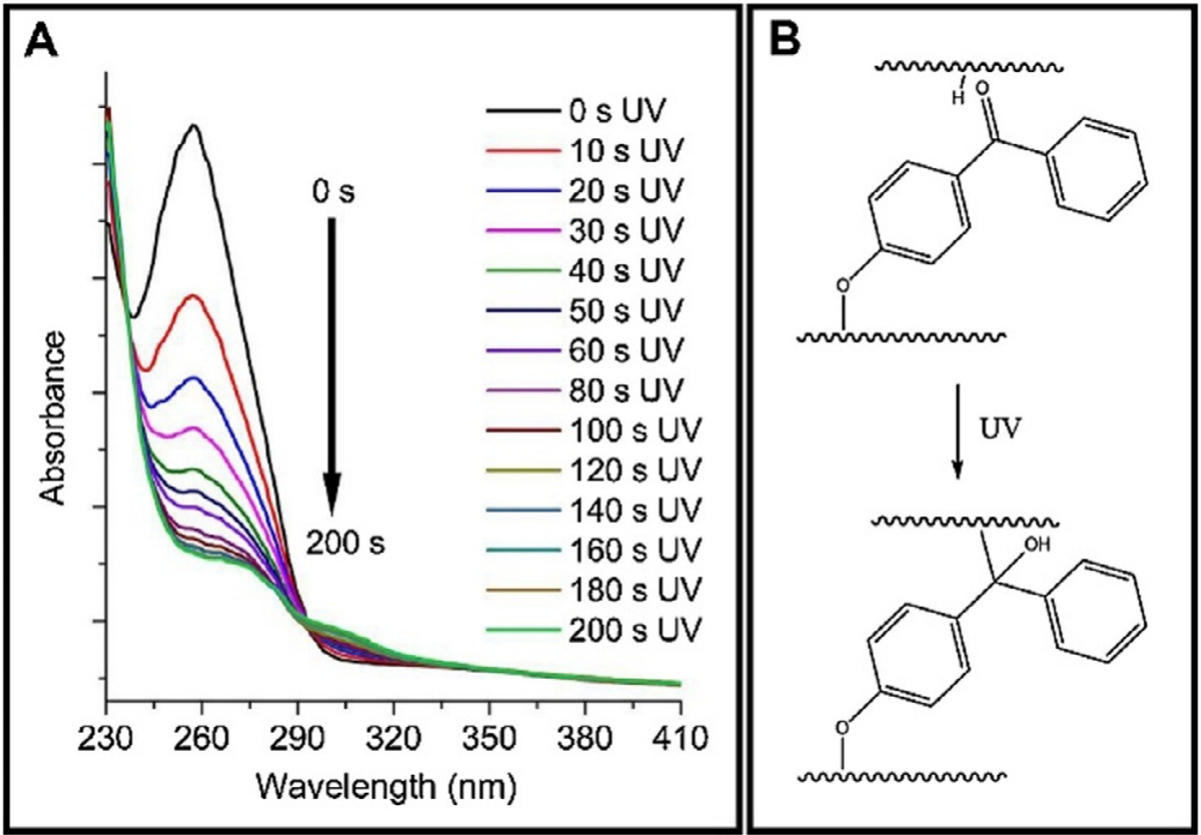
Absorption spectra of benzophenone during UV-curing (A) and the mechanism of benzophenone before and after UV-curing (B). Reprinted from Colloids and Surfaces B: Biointerfaces, Vol. 172, Lin, Z., Goddard, J., Photocurable coatings prepared by emulsion polymerization present chelating properties, pp 143–151, 2018, with permission from Elsevier.

**Fig. 9:**
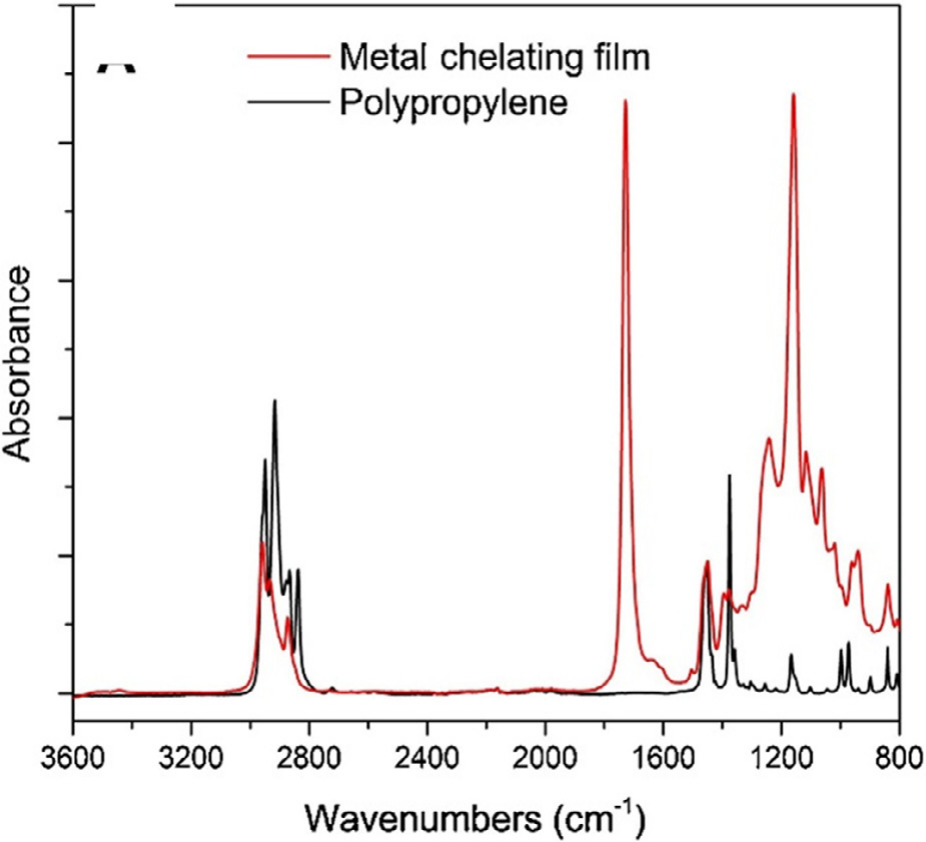
ATR-FTIR spectra for bare polypropylene and the copolymer film. Reprinted from Colloids and Surfaces B: Biointerfaces, Vol. 172, Lin, Z., Goddard, J., *Photocurable coatings prepared by emulsion polymerization present chelating properties*, pp 143–151, 2018, with permission from Elsevier.

**Fig. 10:**
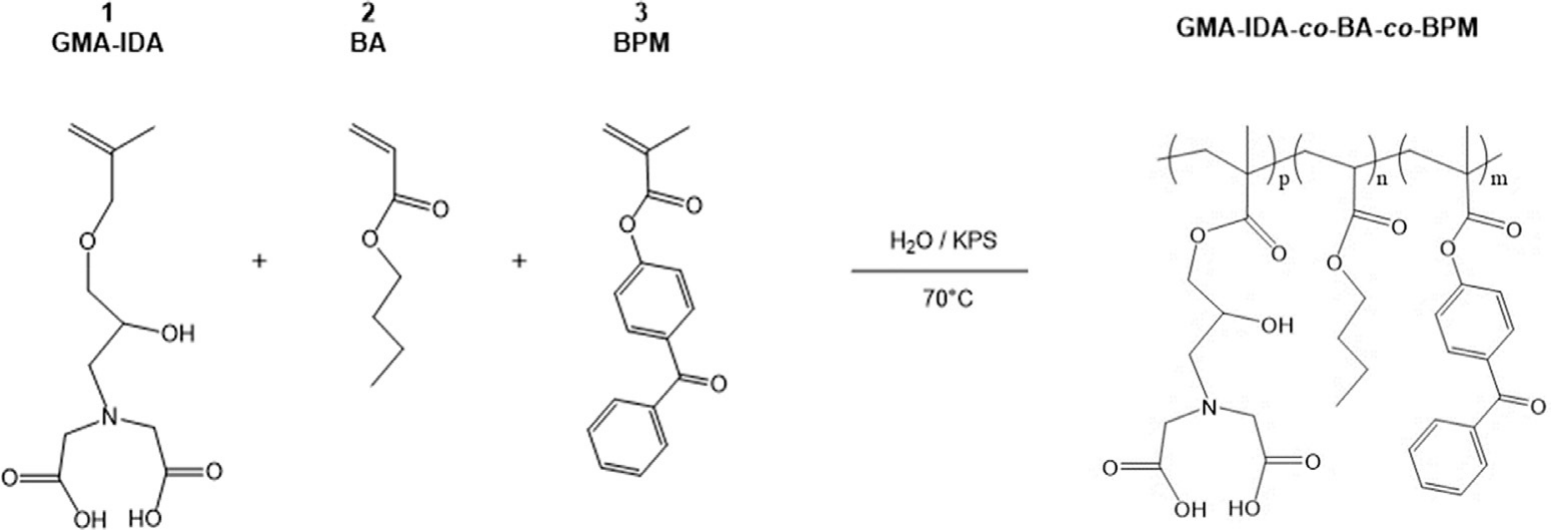
Copolymerization of poly(2-propenoic acid,2-methyl-,3-[*bis*-(*carboxymethyl*) *amino*]−2-hydroxypropyl ester-*co*-*n*-butyl acrylate-*co*-4-benzoylphenyl methacrylate) (GMA-IDA-*co*-BA-*co*-BPM) via emulsion polymerization initiated by potassium persulfate (KPS).

Herein we present a general surfactant-free emulsion polymerization method to synthesize a copolymer consisting of three functional monomers that can be adapted based on the needs of the film. In addition, we provide examples for the functionalization of monomers as building blocks to yield a functional copolymer. Our copolymer includes a glycidyl methacrylate (GMA) monomer functionalized with a metal-chelating iminodiacetic acid (IDA) ligand, *n‑*butyl acrylate (BA), and a UV-curable monomer, 4-benzoylphenyl methacrylate (BPM) as shown in Fig. 10. The stability of this surfactant-free emulsion is imparted by the initiator, KPS. It has been reported that sulfate groups from the persulfate radical species can stabilize a polymer latex without the need for a surfactant [5]. As seen in Fig. 8, the resulting copolymer presents the intended UV-curing functionality and the ATR-FTIR spectra shown in Fig. 9 shows successful copolymerization of poly(n‑butyl acrylate) and GMA functionalized with IDA ligands. Thus, the stability of the emulsion polymerization was sufficient to yield the intended product in this case. Although the presented copolymer shows specific functionalities intended for a specific application in metal chelating, antioxidant active packaging coatings, this method is intended to provide a general emulsion copolymerization process with tips and notes. With slight modifications, the presented methods may be employed to synthesize other functional copolymers via emulsion polymerization.  For example, bio-based monomers, or functionalities such as thermocuring, antimicrobial or non-fouling ligands, or even biocompatible monomers. Functional copolymers have an extensive range of possible applications some of which include food packaging, food safety, water treatment, biomedical implants, or functional polymer coatings. Providing standardized methods lend detailed information on reactor set-ups and method validation that can serve as a foundation for further functional copolymerization syntheses with applications in human health, defense, food and agriculture, and advanced manufacturing.

## Declaration of Competing Interest

The authors declare no conflict of interest.
